# The Influence of Casing-Sand Adhesion on Cementing Bond Strength

**DOI:** 10.1371/journal.pone.0130892

**Published:** 2015-06-26

**Authors:** Xiaofeng Zhao, Zhichuan Guan, Minglei Xu, Yucai Shi, Hualin Liao, Jia Sun

**Affiliations:** 1 College of Petroleum Engineering, China University of Petroleum, Qingdao, Shandong, China; 2 Research Institute of Petroleum Engineering, CNPC Bohai Drilling Engineering Company Limited, Tianjin, China; 3 International Petroleum Service Corporation, SINOPEC, Beijing, China; Duke University Marine Laboratory, UNITED STATES

## Abstract

In the petroleum industry, one of the most serious problems encountered during cementing is the failure at the bonding interface. Many measures including casing-sand adhesion have been developed to improve cementing bond strength. However, due to the lack of detailed study of the technique, many questions remain. The primary goal of this study is to investigate the influence of casing-sand adhesion on cementing bond strength, and to optimize parameters. An orthogonal experiment and a supplementary experiment were conducted. The results indicated that casing-sand adhesion can improve the cementing bond strength. The priority orders of key factors are: sand grain size, sand coverage, adhesive curing temperature and adhesive curing time. The optimal parameters recommended for application are: 1.6mm~1.9mm sand grain size, 60%~70% sand coverage, 30°C curing temperature and 60 hours curing time.

## Introduction

**Fig 1:**
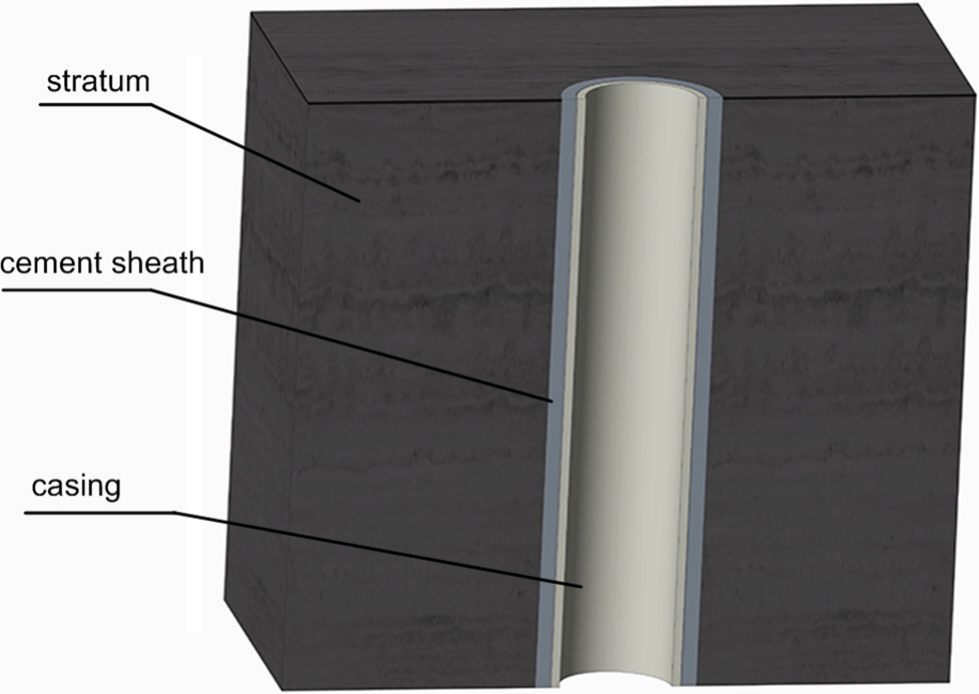
The diagram of oil and gas well.

The cementing process is very important in oil (gas) well construction. Casing and cementing can provide effective zonal isolation of the stratums which penetrated by the wellbore (Fig 1). Leakage behind the casing can reduce the cost effectiveness of the well and may threaten worker safety and the environment. The ability of the casing-cement system to maintain a seal at the casing- cement interface depends on the condition of the casing surface.

A weak casing-cement bond is normally obtained with mill-varnished surface, so in order to alter the surface of casing, a resin-sand coating technique is widely used [1], [2]. This technology can greatly improve the casing-cement bond strength, and has been adopted by many field operators [3]-[5]. Since 1964 many studies have been conducted on casing-sand adhesion, but researchers [1], [2], [6], [7] only reported favorable effects of this technology by brief comparison of bond strength under various casing surface conditions. Even though further study [8], [9] on the mechanism has been carried out, there is no literature addressing the influence of casing-sand adhesion on cementing bond strength and parameter optimization. This paper focuses on the parameters of casing-sand adhesion, and through experimental study, it demonstrates the influence of casing-sand adhesion on cementing bond strength and optimizes parameters. The conclusion of this paper will be one of the supplementary steps for the future study.

## Materials and Methods

### Experiment program design

Interfacial shear strength was selected as the metric of cementing bond strength. The experiment goals were, 1) verify Casing-sand adhesion can improve cementing bond strength as measured by interfacial shear strength under various conditions of casing surface. In this test, casings were dipped into drilling fluid for different durations to simulate different surface conditions; 2) identify the priority orders of factors that influence cementing bond strength and optimize the parameters; 3) investigate the influences of factors. An orthogonal experimental method was selected to reduce the number of trials. Each trial was done twice. If the two results are close to each other, the average point should be obtained; otherwise an additional trial was carried out and the outlier was removed. However, with narrow orthogonal data it is difficult to obtain the detailed influence of factors. Therefore, a supplementary experiment with more factor levels was done.

#### Factor selection

In this study, factors such as sand grain size, sand coverage, adhesive curing time and curing temperature were selected. For the wide variety of adhesive and cement, only one type was selected.

#### Level determination

According to the four factors, L_9_ (3^4^) orthogonal table was selected in this experiment, so, we must determine three levels for each factor.

Previous research of Jilin Oilfield of China [10] showed that the 1.6mm~1.8mm sand grain size and 30/cm^2^ adhere density (equivalent to 60%~76% sand coverage) had more impact in oilfield. Chen’s experiment [4] showed that the maximum interfacial shear strength could reach by 2.4mm sand grain size and 80% sand coverage. From the above researches, the levels of sand grain size are 1.6mm~1.9mm, 2.6mm~2.9mm and 4.0mm~4.5mm and the sand coverage are 30%~40%, 60%~70% and 80%~90%.

**Fig 2:**
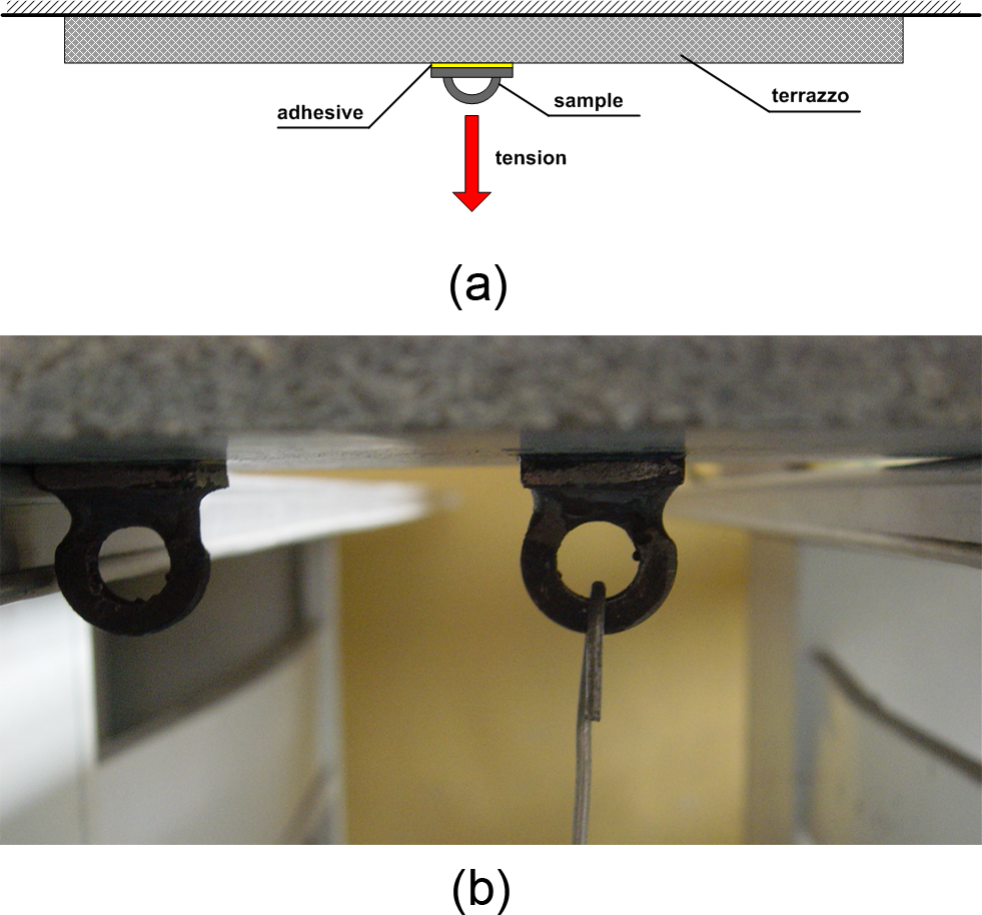
The schematic diagram of testing on the relationship between adhesive curing time and bond strength. (a) schematic diagram. (b) materials.

**Fig 3:**
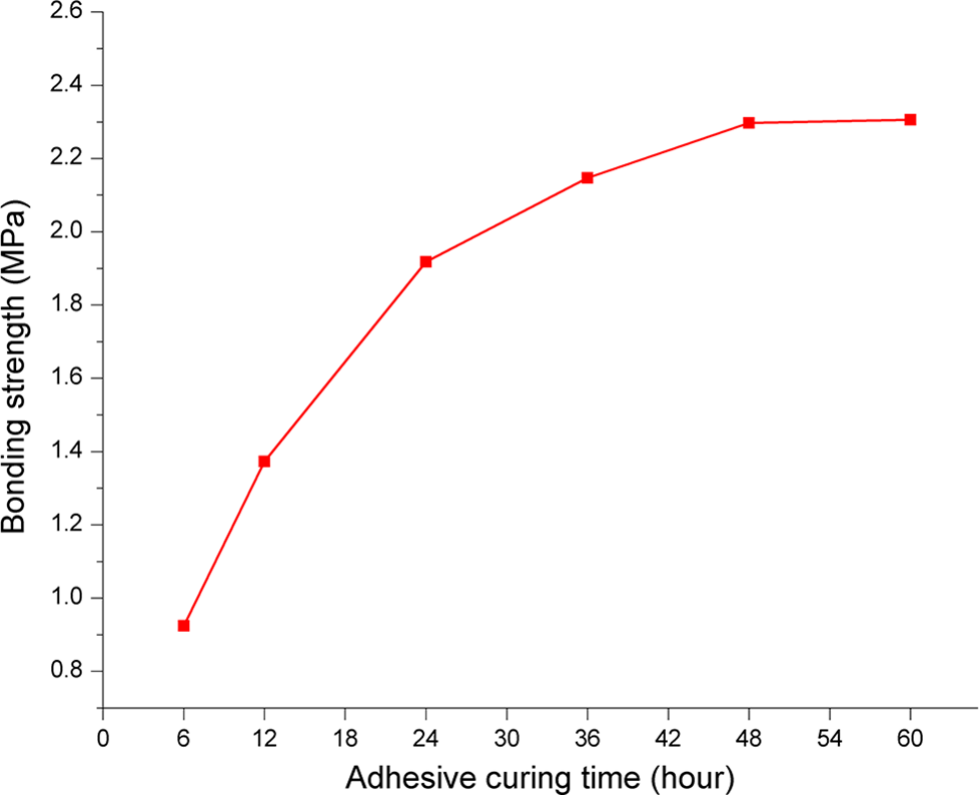
The relationship between adhesive curing time and bonding strength.

A modified bicomponent-epoxy adhesive was adopted in this experiment, which was developed by Shengli Drilling Technology Research Institute of China. The adhesive is made up of epoxy and curing agent, and recommended ratio of epoxy to curing agent is 2.5:1. The merits of the adhesive are low cost, high strength and resistant to heat, water and chemical. In order to determine the levels of adhesive curing time, an additional test has been carried out on the relationship between curing time and bonding strength. In this test, the adhesive was coated on the contact surface of sample and flagstone, and tensile forces which can separate them were measured in different curing time (Fig 2). Each trial was done twice and got the average value. As shown in Fig 3, the adhesive reaches half of the maximum bond strength after 12 hours curing, reaches maximum bond strength after 36 hours curing and no longer increases after 36 hours curing. Some scholars concluded that, running sand-adhering casing before bond strength reaching the maximum could additionally enhance the bond quality. So we determined three levels of adhesive curing time, i.e. 12 hours (half of the maximum bond strength), 36 hours (the maximum bond strength) and 60 hours (fully cured).

Adhesive curing temperature refers to the temperature during periods of sand adhering and cement setting. Actually, measures like heating or water bath are seldom taken in oilfield, because these measures may increase cost and operation difficulty of the technology. So the finally selected curing temperature levels were 0°C, 20°C and 30°C that can be achieved in oilfield.

The levels for this orthogonal experiment are shown in Table 1.

**Table 1:** Levels for orthogonal experiment.

Factors	Sand grain size	Sand coverage	Adhesive curing temperature	Adhesive curing time
Level l	1.6mm~1.9mm	30%~40%	0°C	12 hours
Level 2	2.6mm~2.9mm	60%~70%	20°C	36 hours
Level 3	4.0mm~4.5mm	80%~90%	30°C	60 hours

But for the influence analysis of factors, three levels are not enough, so we added more levels in the supplementary experiment, i.e. 0.8mm~1.2mm sand grain size, four levels for sand coverage including 20%~30%, 40%~50%, 50%~60%, 70%~80%, three levels for adhesive curing time including 24 hours, 48 hours, 120 hours and 40°C adhesive curing temperature.

### Experiment principles and equipment

**Fig 4:**
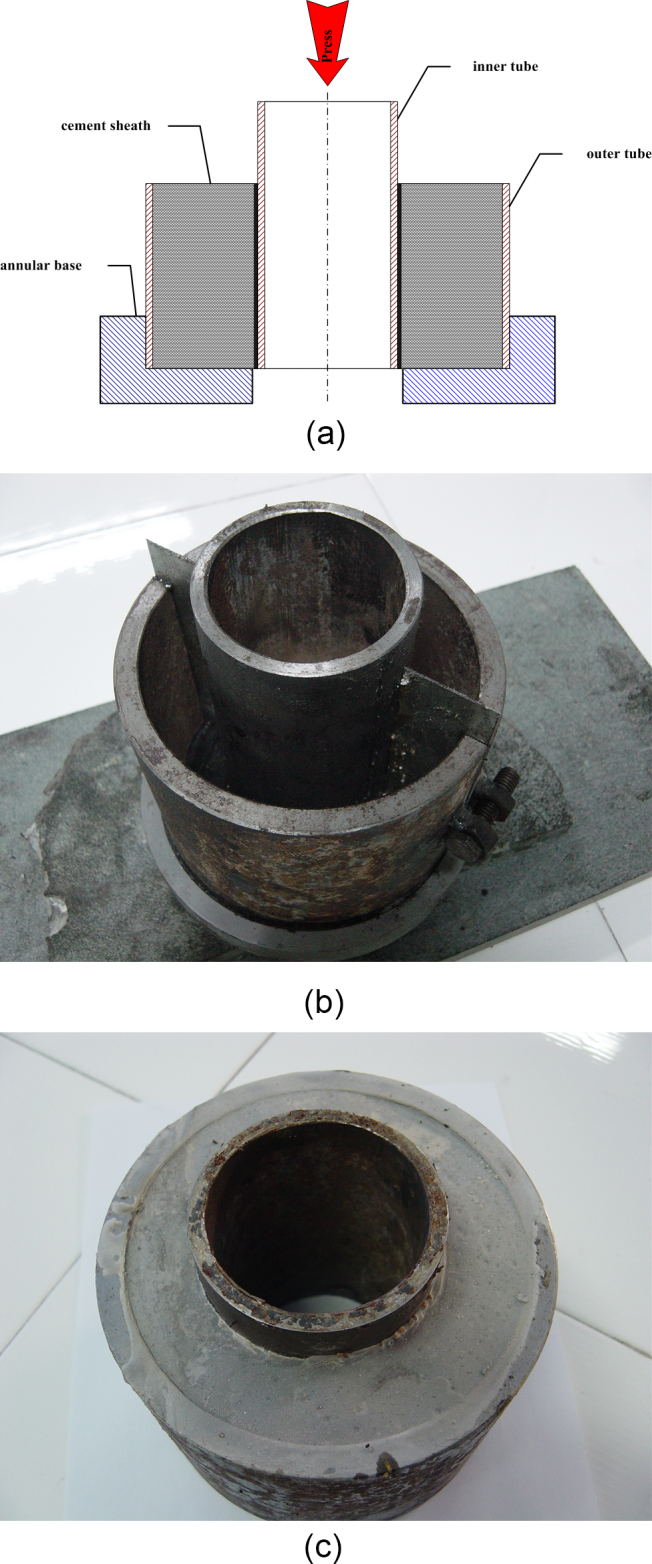
The principle and materials of interfacial shear strength measurement. (a) schematic diagram of experiment. (b) the inner tube, the outer tube and the annular base. (c) the cemented tubes.

**Fig 5:**
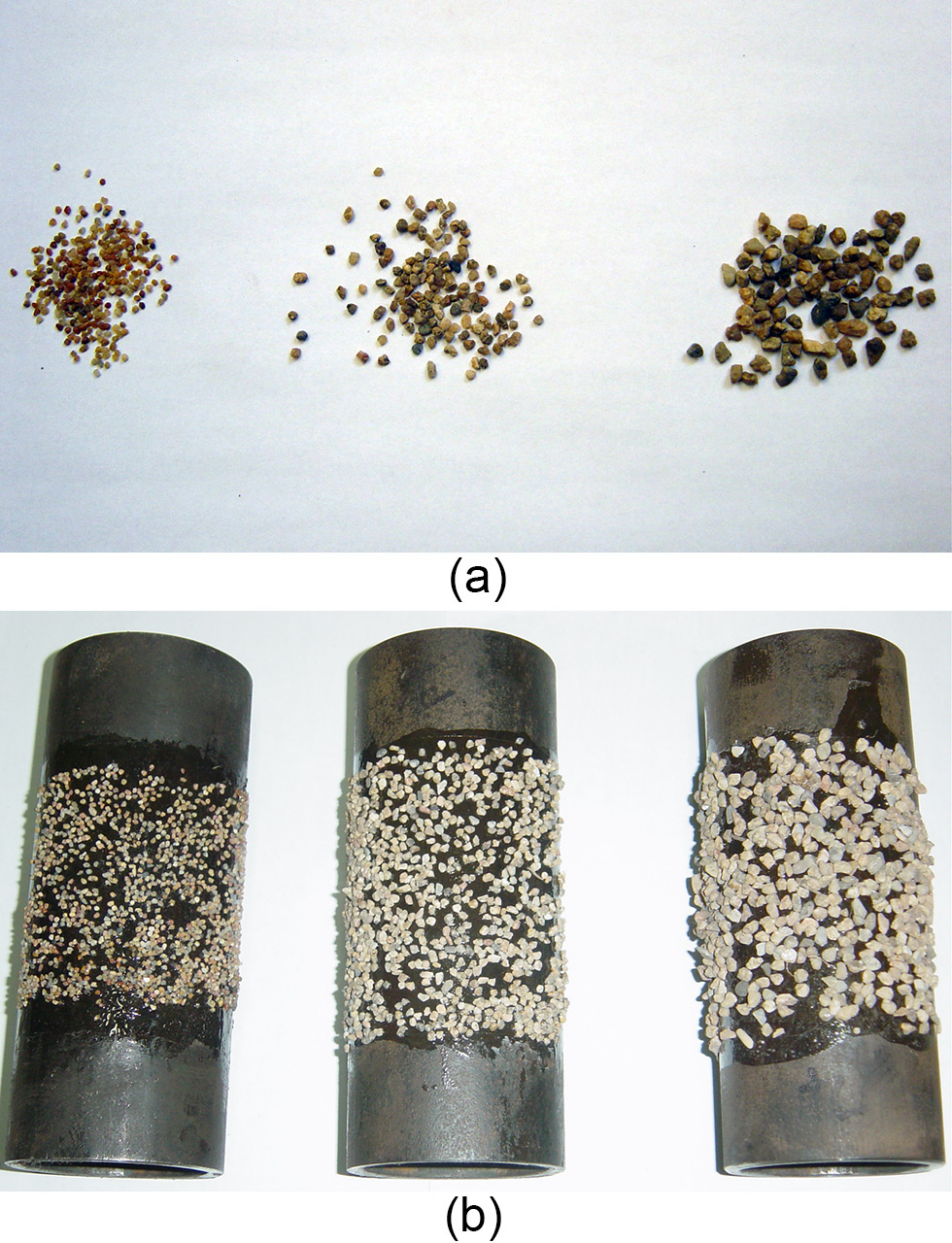
The filtered sand and inner tubes adhered with sand. (a) Filtered building sand. (b) inner tubes adhered with sand of different size.

**Fig 6:**
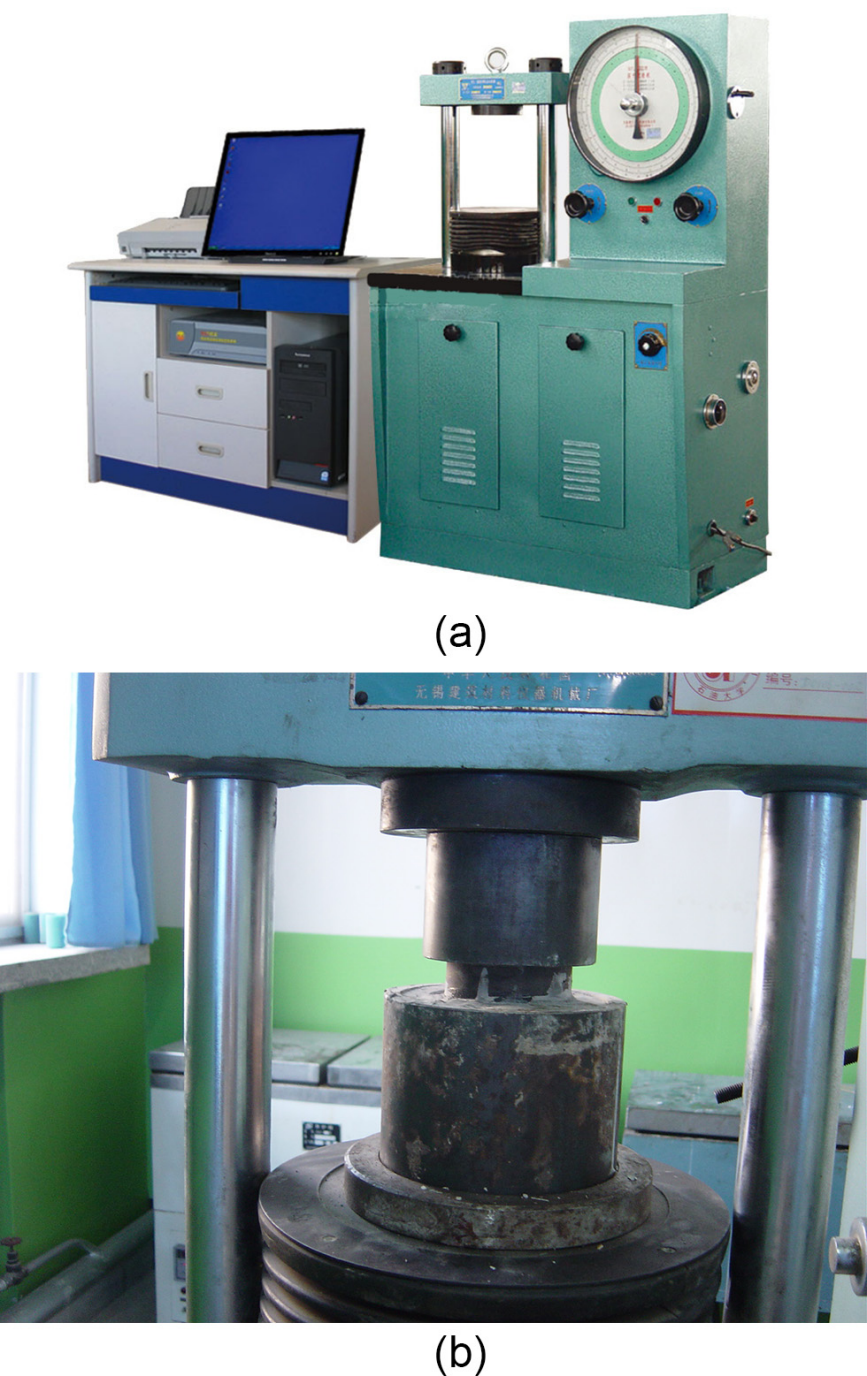
The equipment for experiment. (a) NYL-300 press machine. (b). model put on the press machine.

The interfacial shear strength adopted in this study refers to the shear stress when the casing-cement interface slips. The principles and materials are shown in Fig 4, the model is composed of two cemented tubes and an annular base. The inner and outer tube diameter and height are 73mm, 120mm and 140mm, 100mm. The inner diameter of the annular base is 83mm which is slightly larger than the diameter of inner tube. During the model preparation, the surface of inner tube should be adhered with filtered building sand (Fig 5) and make sure the two tubes were concentric and aligned in one end before cementing. Shengwei G-cement of 0.44 water-cement ratio was injected into the annulus between inner and outer tubes, having time of curing 24 hours at room temperature and normal pressure, another 4 days at 75°C and normal pressure. After cement setting, the model was put on the annular base, and fixed by the groove. Then pressed the model on the press machine (Fig 6B) and record the maximum force when the inner tube slipped. The press machine NYL-300 with maximum range of 300KN adopted is shown in Fig 6A.

## Results and Discussions

**Fig 7:**
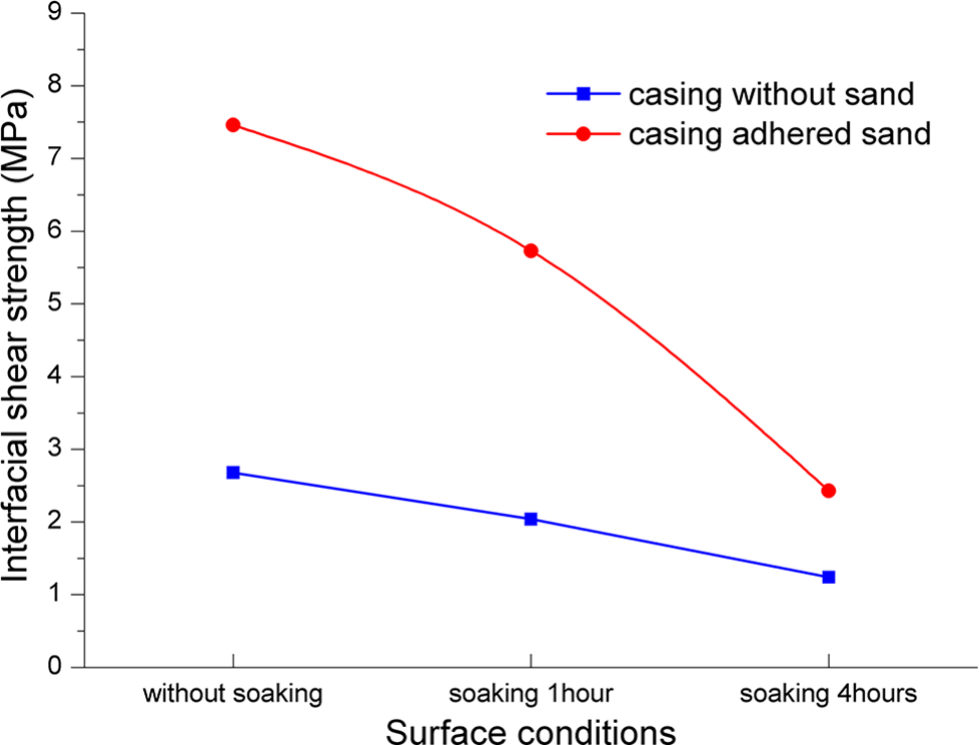
The interfacial shear strength under various conditions of casing surface. The results of casing without sand (blue) and casing adhered sand (red).

Fig 7 shows the testing results of casings being dipped into drilling fluid for different durations. From Fig 7, the shear strength is influenced significantly by the surface condition of casing, and the shear strength reaches the maximum value without the casing dipped into drilling fluid. This is because mud cake was formed at casing surface if it was dipped into drilling fluid, and the longer time dipped, the thicker mud cake is. Meanwhile, the shear strength of sand adhered casings is always higher than the casings without sand, with an average of 1.53 times. Therefore, we conclude that casing-sand adhesion can improve the cementing bond strength effectively.

### Parameters optimization

From the result of the orthogonal experiment and analysis of range, we established the orthogonal table (Table 2).

**Table 2:** The orthogonal table from the result of the orthogonal experiment and analysis of range.

NO.		Sand grain size	Sand coverage	Adhesive curing temperature	Adhesive curing time	Shear strength
1		1	1	1	1	5.73MPa
2		1	2	2	2	7.46MPa
3		1	3	3	3	5.16MPa
4		2	1	2	3	4.16MPa
5		2	2	3	1	5.21MPa
6		2	3	1	2	3.62MPa
7		3	1	3	2	3.23MPa
8		3	2	1	3	5.08MPa
9		3	3	2	1	2.93MPa
level	K_1_	6.117	4.373	4.810	4.623	
	K_2_	4.330	5.917	4.850	4.770	
	K_3_	3.747	3.903	4.533	4.800	
Range		2.370	2.014	0.317	0.177	

#### Range analysis

In orthogonal experiment, range is the difference of level and it reflects the influence of factor on the experiment result. Big range indicates major factor, while small range indicates relatively minor factor. According to the ranges in Table 2, the numerical relationships of the factors are: sand grain size>sand coverage>adhesive curing temperature>adhesive curing time, this means the priority orders of factors are: sand grain size, sand coverage, adhesive curing temperature and adhesive curing time. Among these factors, sand grain size and sand coverage are major factors; adhesive curing temperature and adhesive curing time are minor factors.

#### Level analysis

**Fig 8:**
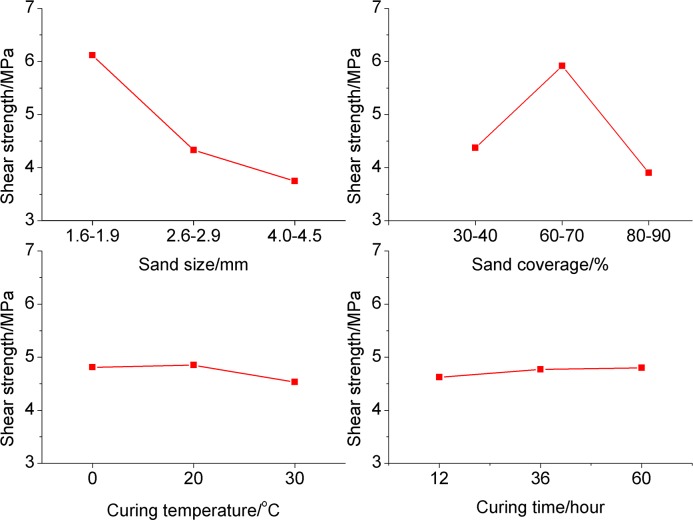
The effect curve of each factor based on the orthogonal experiment result.

According to the principle of orthogonal experiment, the K values of each factor reflect the influence of levels on the result, because K value was obtained while other factors remained unchanged. The effect curves of each factor based on the K values are shown in Fig 8.

From Fig 8, we conclude that the optimal parameters of the four factors are: 1.6mm~1.9mm sand grain size, 60%~70% sand coverage, 20°C adhesive curing temperature and 60 hours adhesive curing time. Better cementing bond quality would be obtained by using the optimized parameters.

### Influences factors

The influences of factors were analyzed based on the result of supplementary experiment.

#### Influence of sand grain size

**Fig 9:**
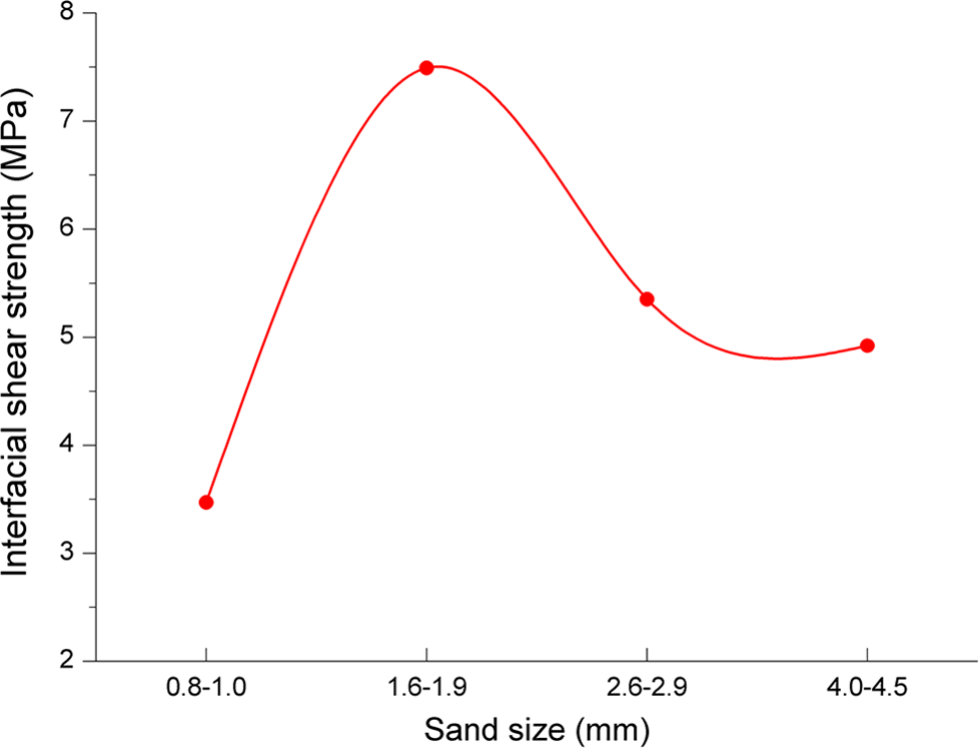
The relationship between sand grain size and interfacial shear strength.

Fig 9 shows the relationship between sand grain size and interfacial shear strength. From Fig 9, the interfacial shear strength reaches the maximum value at data point of 1.6mm~1.9mm, and declines as the sand grain size increases after this point. This is because sands tend to fall off when the sand grain size is too big. However, the interfacial shear strength decreases as the sand grain size decreases before the data point of 1.6mm~1.9mm, this is because sands adhered on casing does not work when the sand grain size is too small.

#### Influence of sand coverage

**Fig 10:**
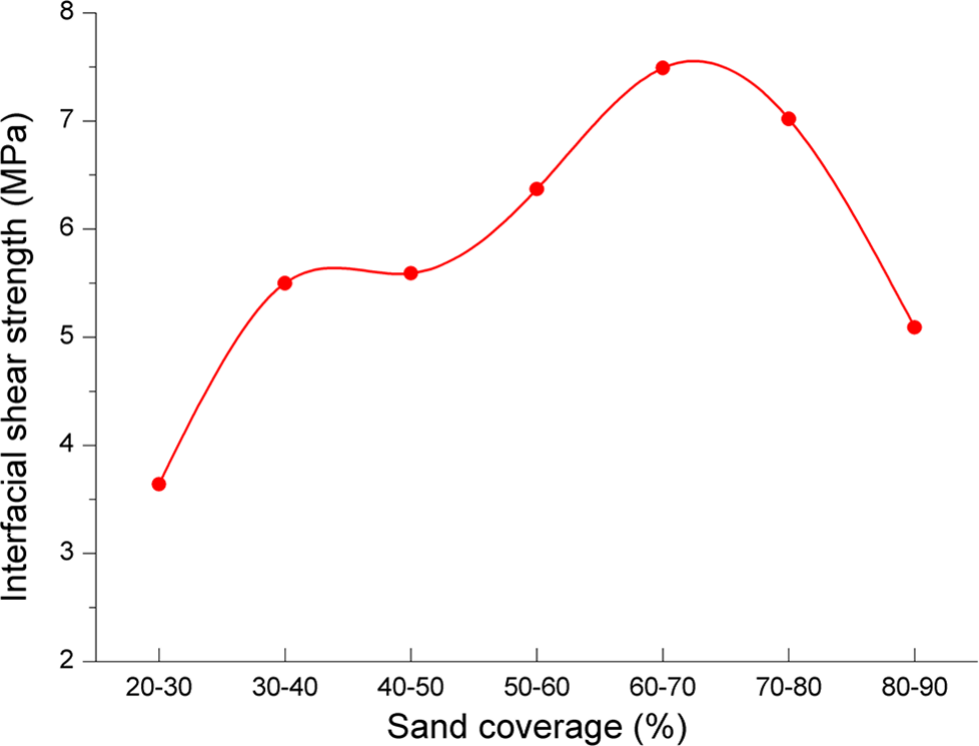
The relationship between sand coverage and interfacial shear strength.

Fig 10 shows the relationship between sand coverage and interfacial shear strength. As shown in Fig 10, interfacial shear strength increases as sand coverage grows and reaches the maximum at data point of 60%~70%, then declines as the sand coverage continues to increase. So we conclude that the interfacial shear strength is not optimal, no matter the sand coverage is too big or small, the casing-sand adhesion would get a better cementing bond quality just when the sand coverage is appropriate.

#### Influence of adhesive curing temperature

**Fig 11:**
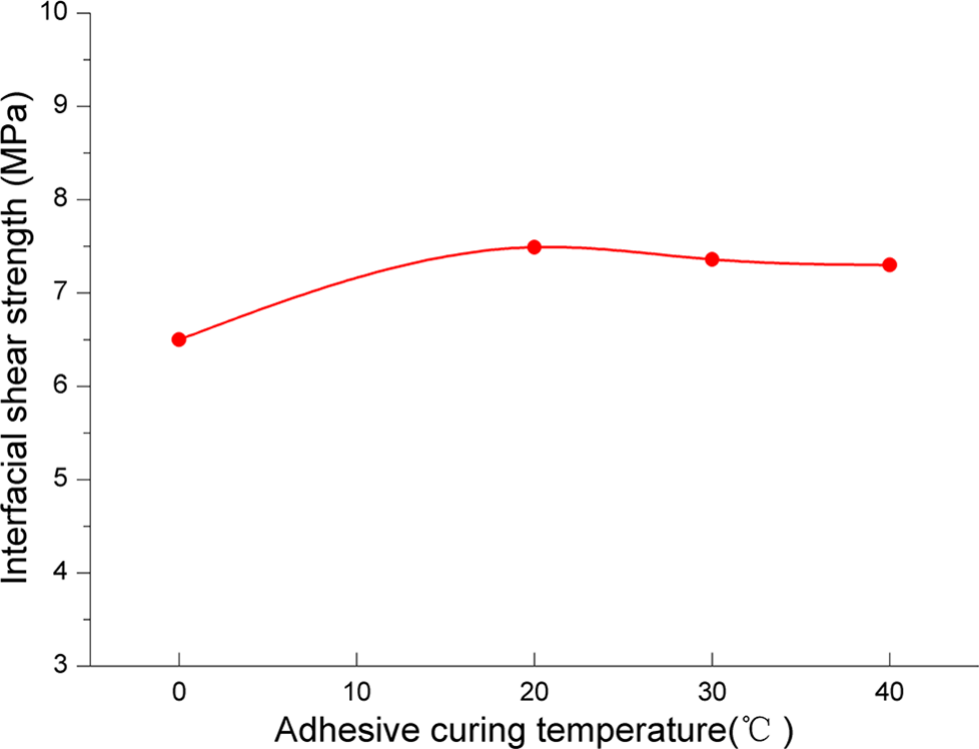
The relationship between adhesive curing temperature and interfacial shear strength.

Fig 11 shows the relationship between adhesive curing temperature and interfacial shear strength. From Fig 11, the curve is steady increase and change small, which indicates that adhesive curing temperature has small influence on interfacial shear strength. The uptrend of interfacial shear strength is evident when the adhesive curing temperature is under 20°C, and it maintains steady when the adhesive curing temperature is above 20°C. So we conclude that adhesive curing temperature has less influence on the interfacial shear strength and the basic temperature of 20°C is acceptable. The controlling of temperature requires high quality of equipment, and if excessively increase the adhesive curing temperature, the economic efficiency will be impacted, so it is better to use the temperature of oilfield.

#### Influence of adhesive curing time

**Fig 12:**
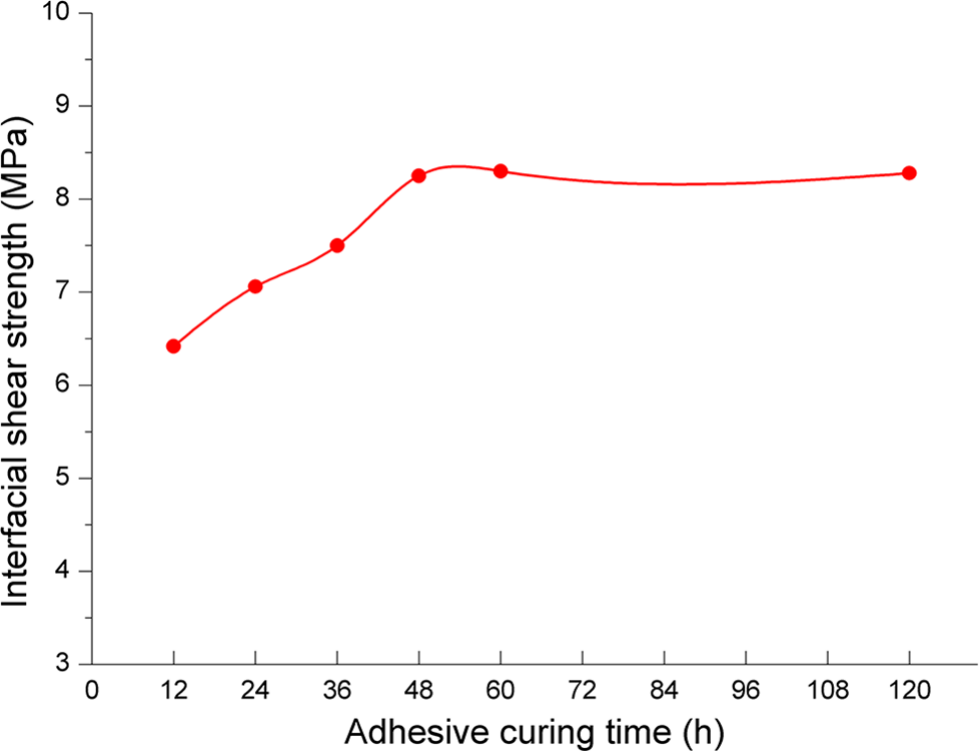
The relationship between adhesive curing time and interfacial shear strength.

Fig 12 shows that the interfacial shear strength increasing slowly as the adhesive curing time increases, and does not change when the adhesive curing time is more than 60 hours. It indicates that if only the adhesive curing time is enough and sand is fully adhered, the cementing bond quality could be better. So in order to eliminate the possible influence of adhesive curing time, we should control the time to more than 60 hours in oilfield application.

## Conclusions

1. The surface conditions of casing have significant effect on cementing bond quality, and casing-sand adhesion can improve the cementing bond strength effectively.
2. The priority orders of factors are: sand grain size, sand coverage, adhesive curing temperature and adhesive curing time. Among these factors, sand grain size and sand coverage are major factors; adhesive curing temperature and adhesive curing time are minor factors.
3. The parameters optimized for field application are: 1.6mm~1.9mm sand grain size, 60%~70% sand coverage, 30°C adhesive curing temperature and 60 hours adhesive curing time.
